# The dark side of the esophagus

**DOI:** 10.4322/acr.2021.284

**Published:** 2021-05-06

**Authors:** Daniela Martins, Rita Marques, Pedro Costa, João Pinto de Sousa

**Affiliations:** 1 Centro Hospitalar de Trás-Os-Montes e Alto Douro, Departamento de Cirurgia Geral, Vila Real, Portugal.

**Keywords:** Acute esophageal necrosis, Black esophagus, Upper gastrointestinal hemorrhage, Ischemia, Endoscopy Gastrointestinal

## Abstract

Acute esophageal necrosis (AEN), also known as “black esophagus,” is an entity characterized by the circumferential black appearance of esophageal mucosa, usually associated with hypoperfusion and gastric outlet obstruction. This entity has a reported prevalence of up to 0.2%, affecting predominantly elderly men with multiple comorbidities. Most cases resolve with conservative treatment with no need of surgical intervention. However, the overall prognosis is poor, with mortality reaching one-third of cases due to the patient’s underlying illness. In this article we present three cases of patients with AEN.

## INTRODUCTION

“Black esophagus,” a term used to mention acute esophageal necrosis (AEN), corresponds to the endoscopic, circumferential, black appearance of the distal esophagus. AEN is a rare condition with a low prevalence and is associated with esophageal hypoperfusion and gastric outlet obstruction. According to some endoscopic series, AEN prevalence ranges between 0.01% and 0.2%. However, AEN is being increasingly recognized as a cause of gastrointestinal (GI) bleeding, and its actual prevalence is thought to be higher due to subclinical presentations and the lack of endoscopic examinations in every critical ill patient.^1^^,^^2^

Male sex, older age, multiple comorbidities (e.g., such as cardiovascular disease, poor nutrition status, immunosuppression, diabetes mellitus, chronic renal failure, chronic lung disease, and alcohol abuse) are some of the risk factors associated with black esophagus. AEN usually presents as GI bleeding. The treatment of the underlying condition, namely (i) nil per os, (ii) fluid therapy, and (iii) proton pump inhibitors, are the basis of the clinical management. Usually, AEN prognosis is poor due to the severity of the underlying disease rather than the black esophagus itself.^2^

This article aims to review AEN and presents three case reports diagnosed at our institution.

## CASE PRESENTATION

### Case 1

A 59-year-old male sought the emergency department (ED) complaining of abdominal and dorsal gnawing pain over the last week, accompanied by dark vomiting on the last day. He presented several dark vomitus at the ED. There was no fever, respiratory or urinary symptoms, or other complaints.

His medical history included type-II diabetes mellitus (DM), surgery to treat a peptic ulcer, and an episode of AEN after a vascular surgery (peripheral critical ischemia) 3 months prior. He denied alcohol abuse, liver disease, or non-steroidal anti-inflammatory drug (NSAID) use.

On physical examination, he was hemodynamically stable, with normal glycemia. The abdomen was thoroughly tender on palpation, without peritoneal irritation signs. Rectal exam revealed melena. The abdomen and thoracic x-rays were normal.

Laboratory workup revealed a hemoglobin level of 11.10 g/dL (reference rang [RR]: 12.0–16.0 g/dL); platelet count and renal function tests were normal. The upper endoscopy revealed a circumferential darkened, ulcerated and friable mucosa of the distal third of the esophagus, consistent with AEN and a duodenal ulcer without active bleeding (Figure 11B).

**Figure 1:**
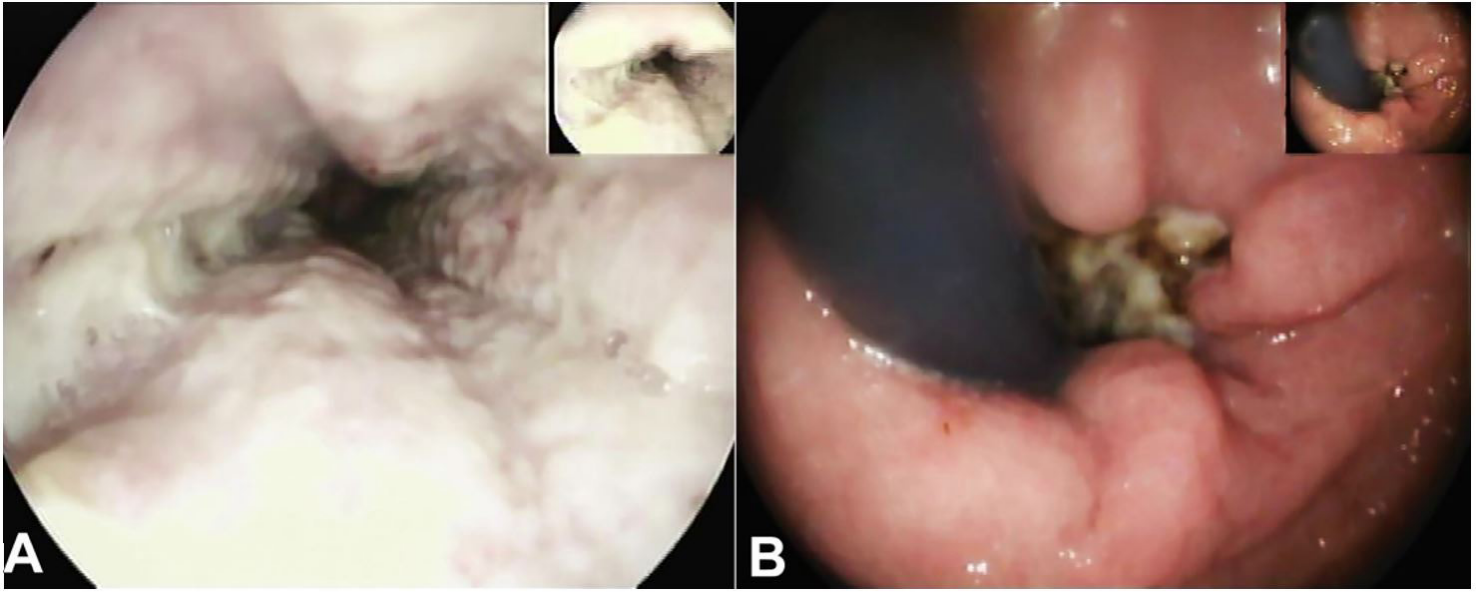
**A** – Upper endoscopy showing ischemic mucosa; **B** – Retrovision of the cardia showing the gastroesophageal transition with an ischemic esophageal mucosa.

There was also a hematic gastric stasis of 1.5L. The patient was submitted to angio-computed tomography (CT), which was normal. His treatment consisted of fluids and proton pump inhibitors (PPI) with alimentary pause.

He had a favorable outcome. The bleeding ceased, and a control endoscopy showed improvement of the necrotic esophageal areas.

### Case 2

An 81-year-old male was brought to the ED with hematemesis and gastric fullness sensation over the last 3 days. He denied stool changes, abdominal pain, or any other symptoms.

His medical history included type-II DM, dyslipidemia, hypertension, and transtibial amputation (1 month before) due to limb ischemia. He had no history of liver disease, NSAID use, or gastroparesis. At the physical exam he was pale, dehydrated, hypotensive and tachycardic, with epigastric pain, but no peritoneal irritation signs.

Laboratory workup revealed a hemoglobin of 9.2g/dL (RR: 12.0–16.0 g/dL) and discreet elevation of inflammatory markers. The upper endoscopy revealed a circumferential ulceration of the medium and distal third of the esophagus, consistent with AEN. Gastric clots without evidence of active bleeding were present (Figure 22B).

**Figure 2:**
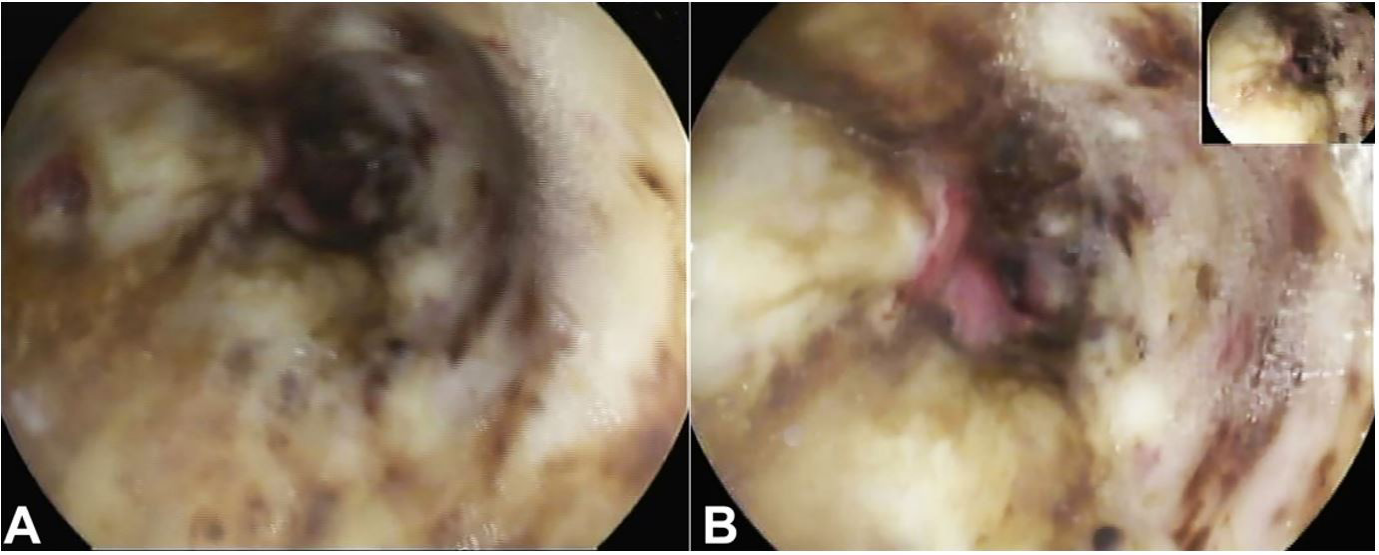
**A** and **B** – Upper endoscopy showing ischemic mucosa.

He was hospitalized and prescribed PPI, intravenous fluids and alimentary pause. His outcome was favorable. The control endoscopy showed an improvement of the necrotic areas. His subsequent appointment revealed no further episodes, with non-symptomatic esophageal stenosis.

### Case 3

A 66-year-old male sought medical care complaining of a continuous and progressive epigastric pain over the last week, associated with vomiting on the last day. No stool changes or other symptoms were referred. His medical history included chronic lung disease, hiatal hernia, and both alcohol and tobacco abuse.

On physical exam he was pale, dehydrated, hypotensive, and tachycardic requiring oxygen supplementation. He presented with epigastric pain with rebound tenderness. The rectal exam was normal.

Laboratory workup revealed normal hemoglobin. However, the inflammatory markers were elevated, and acute renal failure with elevated blood lactates was present. Further blood tests and electrocardiogram excluded myocardial infarction. A thoraco-abdominal CT scan revealed thickening of the middle and lower third of the esophagus, a 3-cm mediastinal collection and a hiatal hernia. Upper endoscopy showed AEN throughout the esophagus without signs of perforation or active bleeding (Figure 33B).

**Figure 3:**
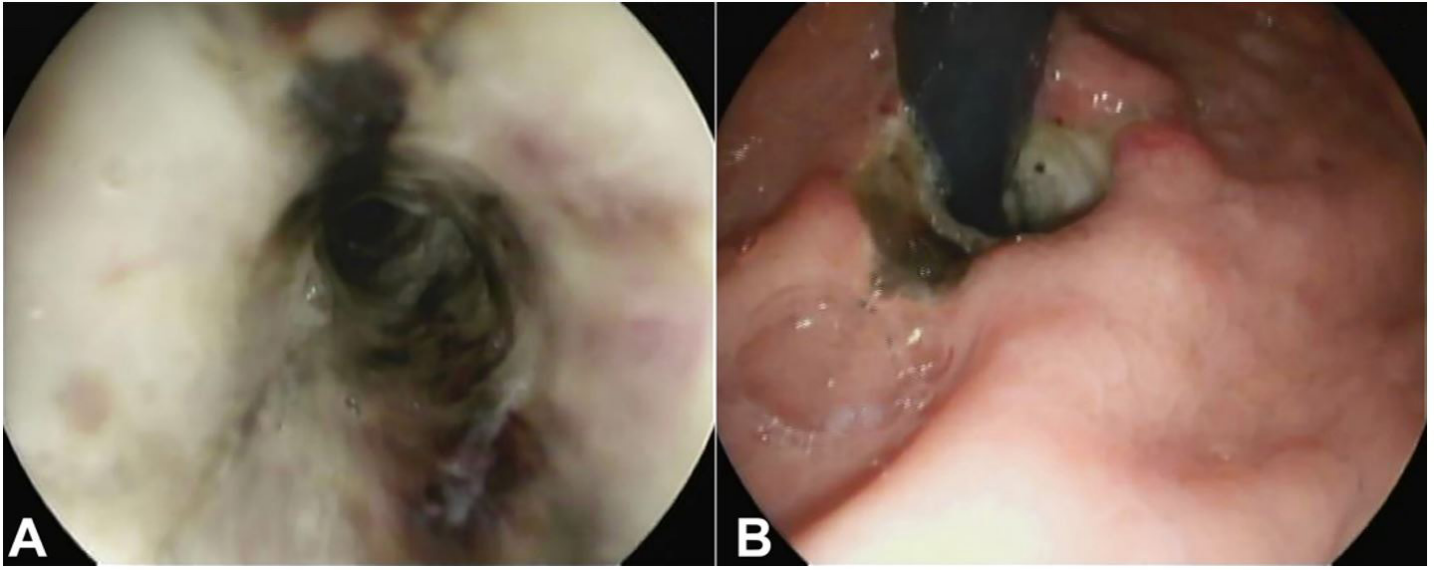
**A** – Upper endoscopy showing ischemic mucosa; **B** – Gastroesophageal transition revealing an ischemic esophageal mucosa.

He was hospitalized and prescribed PPI, fluids, alimentary pause, and antibiotics. Given the risk of esophageal perforation and considering his favorable outcome, a control upper endoscopy was postponed. Nevertheless, a control CT scan showed resolution of the mediastinal collection and a decrease in the esophageal thickening.

The patient was discharged without symptoms. Two months later he was readmitted with a respiratory infection that culminated with his death.

## DISCUSSION

“Black esophagus”, “acute necrotizing esophagitis” or “Gurvits syndrome”, are terms used to describe acute esophageal necrosis, a rare condition characterized by the circumferential black appearance of the mucosa of the esophagus. AEN predominantly affects the distal esophagus, with variable extensions, ending abruptly at the gastroesophageal junction.^1^^,^^3^

The prevalence of AEN ranges from 0.01% to 0.2%, according to one prospective French study by Soussan et. al^4^ and a large retrospective Portuguese case series, by Augusto et al.,^5^ which analyzed clinical and endoscopic data. AEN is more common in men with a mean age of 68 years old.^6^ However, its prevalence might be higher because of its potential subclinical presentation and because it may be present in a critically ill patient, who are not routinely submitted to upper endoscopies. A Japanese study^7^ blamed AEN for 6% of gastrointestinal bleeding.

The pathogenesis of black esophagus is poorly understood, but several mechanisms appear to be involved. The main physio pathologic mechanisms seem to be ischemia (triggered by hypoperfusion states) and gastric obstruction.

The ischemic hypothesis is supported by the poor esophageal vascularization, which renders a fast mucosal injury, particularly in the distal esophagus, which is less vascularized relative to the middle and proximal segments.^2^

On the other hand, the gastric obstruction due to duodenal ulcers, malignancy, or gastric volvulus, promotes the accumulation of gastric contents, which flow back into the esophagus worsening the ischemic injury already settled. Also, AEN is usually associated with duodenal ulcers, which can be explained by the fact that these two structures—the esophagus and the duodenum—receive a common blood supply from branches of the celiac axis.^3^^,^^4^

Vasculopathy (usually present in DM), atherosclerosis, and other cardiovascular diseases or prothrombotic states (present in malignancies), are important conditions that may predispose the patient to tissue ischemia. Malnutrition is another important factor since it reduces the mucosal defenses predisposing the injury.^1^^,^^2^

Other entities, such as the Stevens-Johnson syndrome, alcohol abuse, or local infections (*Klebsiella pneumoniae*; cytomegalovirus; Candida, among others) may also be associated.^2^

AEN usually presents with upper gastrointestinal bleeding (e.g., hematemesis and/or melena) in about 70% of patients. The patients may also present with signs and symptoms of the underlying disease/comorbidities, and usually are very debilitated and malnourished. Vomiting, dysphagia, epigastric or retrosternal pain, and sometimes signs of hypotension and tachycardia are also frequently found.^8^

There are no specific laboratory findings of AEN, which may be associated with the condition itself (e.g., anemia or leukocytosis) or related to an underlying disorder.^2^

AEN is diagnosed by the endoscopic presence of a circumferential friable, ulcerated, dark-colored esophageal mucosa, which predominantly affects the distal third of the organ. The proximal extension is variable, and the mucosa becomes abruptly normal in the gastroesophageal junction. Usually, these endoscopic findings are incidental while investigating upper gastrointestinal bleeding, and contribute to the diagnosis of AEN after ruling out the possibility of the ingestions of corrosive agents.^2^

Mucosal biopsy is not mandatory but confirms the diagnosis. In the presence of dark esophageal mucosa, other entities should be considered; for example, melanoma, melanosis, pseudomelanosis, acanthosis nigricans, melanocytosis of the esophagus, or coal dust deposition.^2^^,^^5^

Currently, there is no standardized treatment of black esophagus. However, fluid reposition, PPI, and alimentary pause are consensual. The treatment also should be directed to the coexisting medical disorders. In cases of sepsis, immune deficiency, rapid decompensation (clinical deterioration, vitals instability) or suspected esophageal perforation, antimicrobial therapy may be warranted.^1^^,^^6^^,^^8^

Patients with AEN have a poor prognosis, resulting from the severity of the comorbidities rather than from the necrotic mucosa lesion. With supportive treatment, mortality reaches 30% and usually is attributed to the underlying disease, while mortality related specifically to AEN is near 6%. Despite the poor prognosis, most cases show endoscopic improvement in 1 week to 1 month.^1^^,^^5^

Black esophagus may lead to acute and long-term complications. The dreadful AEN complication is the esophageal perforation, which occurs early in the disease course, especially when necrosis affects the entire thickness of the esophageal wall. Perforation can reach 7% of cases and should be suspected in association with a rapid deterioration of the patient's status.^9^ In such cases, emergency surgery is indicated. One or 2 weeks after the diagnosis, esophageal strictures—areas of stenosis—may develop, quite often requiring esophageal endoscopic dilatation. Strictures affect between 10% and 25% of patients, and result from scarring tissue as the mucosa heals.^2^^,^^5^

We presented a case series of three patients with AEN. All three patients were men and had several comorbidities (e.g., cardiovascular and ischemic disorders, alcohol abuse, malnourishment, and a peptic ulcer). However, they differ in age and severity of the presentation. Two of them presented with features of GI bleeding, thus implying upper endoscopy. Due to the severity of the case and the absence of GI bleeding history, the third patient initially underwent a CT scan and subsequently an upper endoscopy. All three patients were managed conservatively, with a good response and an absence of esophageal perforation, and were discharged within a week. One of the patients had evidence of esophageal strictures without the need for further dilation. Another patient died months later—not directly to AEN—but due to his poor health state associated with chronic lung disease.

AEN is a rare condition, the endoscopic finding of which is a striking dark appearance of the mucosa, and is associated with a combination of hypoperfusion state and gastric obstruction. It may be incidentally diagnosed when investigating upper GI bleeding in elderly patients with multiple comorbidities and an underlying disease. Despite being rare, black esophagus is a significant cause of GI bleeding, and clinicians should be aware of this condition to enable them to provide adequate management, and be watchful for its possible complications.
